# Different factors modulate visceral and subcutaneous fat accumulation in adults: a single-center study in Brazil

**DOI:** 10.3389/fnut.2025.1524389

**Published:** 2025-02-25

**Authors:** Camila Lima Chagas, Nadja Fernandes da Silva, Isa Galvão Rodrigues, Gabriela Maria Pereira Floro Arcoverde, Victoria Domingues Ferraz, Dário Celestino Sobral Filho, Alcides da Silva Diniz, Cláudia Porto Sabino Pinho, Poliana Coelho Cabral, Ilma Kruze Grande de Arruda

**Affiliations:** ^1^Universidade Federal de Pernambuco - UFPE, Recife, Brazil; ^2^Universidade de Pernambuco - UPE, Recife, Brazil; ^3^Pronto Socorro Universitário Cardiológico de Pernambuco – PROCAPE, Recife, Brazil

**Keywords:** abdominal fat, subcutaneous fat, body fat distribution, obesity, body composition

## Abstract

**Background:**

Abdominal adipose tissue consists of visceral and subcutaneous fat deposits, each with unique metabolic and functional properties. Identifying the characteristics that influence different obesity phenotypes can support targeted prevention and intervention strategies.

**Objective:**

To identify predictive factors associated with visceral and subcutaneous adipose tissue accumulation.

**Methods:**

This is a cross-sectional study including adults of both sexes aged ≥20 years under outpatient care in a public healthcare service in Northeast Brazil. Visceral adipose tissue (VAT) and subcutaneous adipose tissue (SAT) were measured via ultrasound. Anthropometric, clinical, sociodemographic, and behavioral variables were incorporated into the predictive model.

**Results:**

A total of 347 individuals were included. They were median age of 47.0 years (interquartile range: 39.0 to 56.0). Visceral obesity was found in 79.3% of the sample. Adjusted analysis demonstrated that physical inactivity (OR 2.3; 95% CI 1.1–4.7; *p* = 0.023) and elevated waist circumference (WC) (OR 6.4; 95% CI 2.6–15.8 *p* < 0.001) were associated with VAT accumulation. Alcohol consumption increased the likelihood of SAT accumulation by 2.2 times (95% CI 1.3–3.7; *p* = 0.005), while elevated WC raised this likelihood by 4.5 times (95% CI 2.1–9.8; *p* < 0.001). The VAT/SAT ratio was significantly higher in older adults (OR 5.5; 95%CI 2.0–14.8; *p* = 0.001), among individuals of Mixed Race and Black, those with lower educational levels (OR 2.4; 95%CI 1.1–5.2; *p* = 0.028), and in diabetics (OR 2.4; 95%CI 1.2–4.9; *p* = 0.017).

**Conclusion:**

Distinct factors influence visceral and subcutaneous obesity. Sedentary behavior emerged as an independent predictor of visceral obesity, while alcohol consumption was associated with a subcutaneous obesity pattern. Diabetes and sociodemographic factors (older age, non-White race, and lower education) were predictive of an elevated VAT/SAT ratio.

## 1 Introduction

Obesity is a chronic, complex, and multifactorial condition involving biological, psychosocial, socioeconomic, and environmental factors. Obesity can result from high intake of poor-quality foods combined with low energy expenditure (1). Obesity is a serious public health challenge; in recent decades, its prevalence has tripled worldwide, emerging as a major cause of mortality and disability with significant impact on adult morbidity patterns. According to estimates from the World Health Organization (WHO), 2.5 billion adults were classified as overweight in 2022, and in the previous year, obesity contributed to approximately 2.8 million deaths due to non-communicable diseases (NCDs) (2, 3).

Adipose tissue serves multiple functions, primarily as an energy reservoir (4, 5). However, body fat distribution is an additional crucial predictor of cardiovascular risk (4, 5). Abdominal fat comprises subcutaneous and visceral fat, each of which poses distinct risks for metabolic and hemodynamic changes (6). Roughly 80% of total body fat is located subcutaneously, predominantly in the gluteofemoral region, back, and anterior abdominal wall. Visceral fat, however, constitutes 10–20% of total fat in men and 5–8% in women (7) and is located around the viscera and peritoneum, bordering the dorsal side of the intestine and the ventral surface of the kidney (4).

Subcutaneous adipose tissue (SAT) and visceral adipose tissue (VAT) differ in morphology and function. SAT drains venously through the systemic circulation, whereas VAT is perfused by the portal circulation (8, 9), rendering VAT metabolically more active and a greater source of inflammatory cytokines (4, 7, 10). Consequently, the lipolytic activity of abdominal VAT constantly releases free fatty acids, which, drained by the portal vein, accumulate in the liver, leading to alterations in lipid and glucose metabolism (7) and directly contributing to the rise in chronic diseases (6, 11). However, the absolute VAT quantification individually may not fully reflect an individual’s risk for visceral obesity. The VAT/SAT ratio is considered as an alternative and appropriate indicator, as it reflects both visceral fat accumulation and an individual’s predisposition to preferentially store fat viscerally (12, 13). This ratio has also been described as a proxy for cardiometabolic risk (13).

The deleterious effects of excessive body fat are extensive; however, the tendency to store fat in different locations under conditions of excess energy intake varies greatly between individuals (14). Despite this, there remain significant gaps in understanding the profiles of individuals at higher risk of accumulating abdominal fat in either subcutaneous or visceral deposits. Therefore, the objective of this study was to assess the predictive factors influencing the accumulation of subcutaneous and visceral adipose tissue. This approach potentially contributes to identifying characteristics that define subcutaneous and visceral abdominal obesity phenotypes, guiding targeted intervention strategies.

## 2 Materials and methods

This is a cross-sectional study conducted at a public university hospital specializing in cardiology in Northeastern Brazil. Volunteers of both genders aged ≥20 years, receiving outpatient care at a public university hospital in Northeast Brazil, were included. At this outpatient clinic, the population is predominantly composed of individuals with chronic non-infectious diseases: systemic arterial hypertension, diabetes mellitus, metabolic syndrome, and dyslipidemia.

All patients provided informed consent and the study protocol adhered to ethical standards for research involving human subjects and was approved under protocol number 4.659.262/2021. Exclusion criteria were individuals with hepatomegaly and/or splenomegaly, ascites, recent abdominal surgery, underwent surgical weight loss treatment, pregnancy, or had given birth within 6 months prior to the study screening. Participants with physical limitations precluding anthropometric measurements were also excluded, as these conditions could affect intra-abdominal fat measurement.

Sample size was calculated using the STATCALC module in Epi Info software, version 6.04 [WHO/CDC, Atlanta, GA, United States], based on a visceral obesity prevalence of 68.3%, obtained from a pilot study involving the first 30 patients. A 5.0% standard error and a 95% confidence interval were applied, resulting in a minimum required sample of 333 patients. To account for potential losses, the sample size was increased by 10%, leading to a final sample size of 367 individuals.

VAT and SAT were assessed using ultrasound (US) with a Vivid T8 Pro Color Doppler Ultrasound System (GE, P.O., Asia). All participants were evaluated in the supine position, with the right arm raised and a minimum fasting period of 4 h (15, 16). A 3.5 MHz convex electronic transducer was positioned transversely 1 cm above the umbilical scar. Visceral fat thickness was defined as the greatest distance, in centimeters, between the inner (deep) surface of the rectus abdominis muscle and the anterior wall of the aorta. Subcutaneous fat thickness was measured as the distance, in centimeters, between the skin and the upper surface of the rectus abdominis muscle (16). Measurements were taken with participants exhaling and without abdominal pressure to avoid underestimation. Each measurement was taken twice and repeated if the measurement error exceeded 0.1 cm (16).

Cut-off values for visceral obesity were set at VAT ≥5.39 cm for men and ≥ 4.27 cm for women (17). Subcutaneous obesity was defined by SAT values above the upper tertile for each sex, with thresholds of ≥2.83 cm for men and ≥ 3.68 cm for women (18). The visceral-to-subcutaneous fat ratio (VAT/SAT) was calculated, with values above the highest tertile for each sex (≥3.60 for men and ≥ 2.14 for women) used as indicators of visceral fat accumulation predisposition and as criteria for elevated VAT/SAT ratio. Among anthropometric parameters, body mass index (BMI) was calculated using the equation weight/height^2^, following WHO classifications (19). Waist circumference (WC) was measured at the narrowest point between the last rib and the iliac crest (20) and classified as WC ≥94 cm for men and ≥ 80 cm for women to indicate higher values (19).

Weight (kg) and height (m) were measured according to techniques recommended by Lohman et al. (20), using an electronic scale (Welmy^®^, Santa Bárbara d’Oeste, São Paulo, Brazil) with a capacity of 150 kg and an accuracy of 100 g, equipped with an attached stadiometer precise to 1 mm. For WC (cm) measurement, a flexible, non-elastic measuring tape with 0.1 cm accuracy was used. Measurements were collected twice by trained observers, with additional measurements taken if discrepancies greater than 1 cm or 100 g were noted.

Sociodemographic variables included age, sex, self-reported race & ethnicity (categorized as White, Black, and Mixed race), years of education, per capita family income, and socioeconomic status. Clinical variables included hypertension and diabetes mellitus, defined by prior medical diagnosis, use of antihypertensive, oral hypoglycemic, or insulin medications, and recorded in the participant’s medical records. To determine socioeconomic status, the “Brazilian Economic Classification Criteria” established by the Brazilian Association of Anthropology and the Brazilian Association of Research Companies (21) was used. This tool scores household possessions and the education level of the family head, classifying individuals into economic classes A1, A2, B1, B2, C1, C2, D, and E, from highest to lowest purchasing power (21). These economic classes were subsequently recategorized into high (A1 and B1), middle (B2 and C1), and low (C2, D, and E) socioeconomic status (22).

Behavioral variables included alcohol consumption, smoking, and physical activity level. Smoking status was categorized as smoker (individual currently smoking), non-smoker (individual who never smoked or quit over 10 years ago), and former smoker (individual who quit between one and 10 years before the study) (23). Alcohol consumption was recorded as a dichotomous variable (“yes” or “no”). To assess physical activity, the International Physical Activity Questionnaire (IPAQ) short version was used, covering four domains of physical activity: leisure, household activities, occupational activities, and transportation-related activities. A physical activity score in minutes per week was calculated by summing the time spent on all activities, with <150 min per week serving as the cutoff for classifying individuals as insufficiently active or sedentary (24, 25).

Data were analyzed using the Statistical Package for Social Sciences – SPSS version 13.0 (IBM^®^ Inc., Chicago, IL, United States). Continuous variables were tested for normality using the Kolmogorov–Smirnov test. Variables with a normal distribution were described using mean and standard deviation, while non-Gaussian variables were presented as medians and interquartile ranges. Proportions were reported with a 95% confidence interval using a binomial approximation to the normal distribution. Intra- and inter-observer reproducibility of the US measurements was evaluated using the intraclass correlation coefficient and 95% limits of agreement, with triplicate measurements for each anatomical site.

A univariate analysis was conducted between the dependent variables (visceral and subcutaneous obesity) and the independent variables, using the Chi-square test and determining prevalence ratios (PR) with their 95% confidence intervals. To examine the independence of associations between dependent and independent variables, a multivariable binary logistic regression model was developed. Independent variables were tested for multicollinearity using Variance Inflation Factor (VIF) and Tolerance statistics (>0.10 and < 3). Logistic regression was performed using the purposeful selection method, where variables associated with the outcome with *p* < 0.20 in univariate analysis were included. Equality of hypotheses was rejected for *p* < 0.05.

## 3 Results

Intra- and inter-observer calibration evaluation for US procedures demonstrated a high inter-observer reproducibility, with Intraclass Correlation Coefficients (ICC) exceeding 0.97 for VAT and greater than 0.98 for SAT. Intra-observer reproducibility was also high, with ICC values above 0.90 for all VAT and SAT assessments.

A total of 367 patients were initially screened. Following exclusions (data inconsistencies or refusal), the final sample included 347 individuals. They were most women (66.3%), with a median age of 47.0 years (interquartile range: 39.0–56.0). A total of 78.8% of the sample presented with more than 9 years of education, and 62.0% classified as middle to low socioeconomic status. Hypertension and diabetes mellitus was found in 47.3 and 25.1%, respectively. Around 75% of the participants were insufficiently active, and 92.0% were overweight by BMI. The prevalence of visceral obesity was 79.3% (Table 1).

**Table 1:** Characteristics of the sample (*N* = 347).

Variables	N	%	95% CI
Sex			
Male	117	33.7	29.0–38.9
Female	230	66.3	61.2–71.1
Age in years			
20–59	298	85.9	81.8–89.2
≥60	49	14.1	10.9–18.2
Race and ethnicity			
White	113	32.6	27.9–37.7
Black	75	21.6	17.6–26.2
Mixed race	159	45.8	40.6–51.1
Years of study			
≤9	76	22.0	17.9–26.6
>9	270	78.8	73.2–81.9
Income			
Lowest tertile	230	66.5	61.2–71.1
2nd and 3rd tertile	116	33.5	28.7–38.6
Social status			
A1 e B1 (High)	24	7.1	4.7–10.1
B2 e C1 (Middle)	183	53.8	47.5–57.9
C2. D e E (Low)	133	39.1	33.4–43.6
SAH	164	47.3	42.1–52.5
DM	87	25.1	20.8–29.9
Alcohol intake	153	44.1	39.0–49.4
Physical activity status			
Insufficient (<150 min/week)	251	74.9	67.4–76.8
Sufficient (≥150 min/week)	87	25.1	20.8–29.9
Smoking status			
Current	11	3.2	1.8–5.6
Non-smoker	271	78.1	73.4–82.1
Former	65	18.7	15.0–23.2
BMI (kg/m^2^)			
Underweight	50	14.4	11.1–18.5
Normal range	82	23.6	19.5–28.4
Excess weight	215	62.0	56.8–66.9
Waist circumference			
Normal	115	33.4	28.4–38.2
Elevated	229	66.6	60.9–70.8
Visceral obesity			
No	72	20.7	16.8–25.3
Yes	275	79.3	74.7–83.2

95% CI: 95% confidence interval; BMI: body mass index; DM: Diabetes mellitus; SAH: systemic arterial hypertension.

Univariate analyses are detailed in Tables 2–4. Visceral obesity was associated with female sex, age ≥ 60 years, lower education level, higher income, lower social class, hypertension, diabetes, physical inactivity, smoking (current or former), overweight, and elevated waist circumference. Higher subcutaneous fat accumulation was associated with age (more common in individuals aged 20–59), higher education, absence of diabetes, alcohol consumption, normal weight, and elevated WC. An elevated VAT/SAT ratio was associated with older age, lower education level, higher income, hypertension, diabetes, current or former smoking, overweight, elevated waist circumference, and abstention from alcohol consumption.

**Table 2:** Factors associated with visceral adiposity among outpatients from a university cardiology hospital (*N* = 347).

Variables	Visceral obesity				PR (95 CI%)	*P*
	No		Yes			
	n	%	n	%		
Sex						0.031
Male	32	27.4	85	72.6	0.88 (0.78–0.99)	
Female	40	17.4	190	82.6	1.0	
Age in years						0.002
20–59	70	23.5	228	76.5	1.0	
≥60	2	4.1	47	95.9	1.25 (1.15–1.37)	
Race and ethnicity						0.862
White	25	22.1	88	77.9	1.0	
Black	16	21.3	59	78.7	1.01 (0.87–1.18)	
Mixed race	31	19.5	128	80.5	1.03 (0.91–1.17)	
Years of study						<0.001
≤9	2	2.6	74	97.4	1.30 (1.21–1.42)	
>9	69	25.6	201	74.4	1.0	
Income						0.003
Lowest tertile	58	25.2	172	74.8	0.84 (0.76–0.93)	
2nd and 3rd tertile	13	11.3	102	88.7	1.0	
Social status						0.011
A1 e B1 (High)	9	37.5	15	62.5	1.0	
B2 e C1 (Middle)	43	23.5	140	76.5	1.22 (0.89–1.69)	
C2. D e E (Low)	18	13.6	114	86.4	1.38 (1.01–1.90)	
SAH						<0.001
No	57	31.1	126	68.9	1.0	
Yes	15	9.1	149	90.9	1.32 (1.18–1.47)	
DM						0.001
No	65	25.0	195	75.0	1.0	
Yes	7	8.0	80	92.0	1.22 (1.12–1.35)	
Alcohol intake						0.738
No	39	20.1	155	79.9	1.0	
Yes	33	21.6	120	78.4	0.98 (0.88–1.09)	
Physical activity status						0.002
Insufficient (<150 min/week)	42	16.7	209	83.3	1.24 (1.06–1.44)	
Sufficient (≥150 min/week)	29	32.6	60	67.4	1.0	
Smoking						0.019
Current	1	9.1	10	90.9	1.20 (0.98–1.46)	
Non-smoker	65	24.0	206	76.0	1.0	
Former	6	9.2	59	90.8	1.19 (1.08–1.32)	
BMI (kg/m^2^)						<0.001
Underweight	14	28.0	36	72.0	1.11 (0.88–1.41)	
Normal range	29	35.4	53	64.6	1.0	
Excess weight	29	13.5	186	86.5	1.34 (1.13–1.58)	
Waist circumference						<0.001
Normal	51	44.3	64	55.7	1.0	
Elevated	20	8.7	209	91.3	1.64 (1.39–1.94)	

95% CI: 95% confidence interval; BMI: body mass index; DM: Diabetes mellitus; PR: prevalence ratio; SAH: systemic arterial hypertension.

**Table 3:** Factors associated with subcutaneous adiposity among outpatients from a university cardiology hospital (*N* = 347).

Variables	Subcutaneous obesity				PR (95% CI)	P
	No		Yes			
	n	%	n	%		
Sex						0.957
Male	78	66.7	39	33.3	1.0	
Female	154	67.0	76	33.0	1.0 (0.72–1.36)	
Age in years						<0.001
20–59	186	62.4	112	37.6	1.0	
≥60	46	93.9	3	6.1	0.16 (0.05–0.47)	
Race and ethnicity						0.200
White	82	72.6	31	27.4	1.0	
Black	51	68.0	24	32.0	1.17 (0.75–1.82)	
Mixed race	99	62.3	60	37.7		
Years of study						0.023
≤9	59	77.6	17	22.4	0.62 (0.39–0.96)	
>9	172	63.7	98	36.3	1.0	
Income						0.331
Lowest tertile	81	70.4	34	29.6	0.88 (0.63–1.23)	
2nd and 3rd tertile	150	65.2	80	34.8	1.0	
Social status						0.042
A1 e B1 (High)	19	79.2	5	20.8	1.0	
B2 e C1 (Middle)	112	61.2	71	38.9	1.86 (0.84–4.15)	
C2. D e E (Low)	96	72.7	36	27.3	1.31 (0.57–3.0)	
SAH						0.222
No	117	63.9	66	36.1	1.0	
Yes	115	70.1	49	29.9	0.83 (0.61–1.22)	
DM						0.001
No	161	61.9	99	38.1	1.0	
Yes	71	81.6	16	18.4	0.48 (0.30–0.77)	
Alcohol intake						<0.001
No	151	77.8	43	22.2	1.0	
Yes	81	52.9	72	47.1	2.12 (1.55–2.90)	
Physical activity status						0.074
Insufficient (<150 min/week)	66	74.2	23	25.8	0.71 (0.48–1.05)	
Sufficient (≥150 min/week)	160	63.7	91	36.3	1.0	
Smoking						0.881
Current	7	63.6	4	36.4	1.12 (0.50–2.49)	
Non-smoker	183	67.5	88	32.5	1.0	
Former	92	80.0	23	20.0	0.62 (0.41–0.92)	
BMI (kg/m^2^)						<0.001
Underweight	44	88.0	6	12.0	0.25 (0.11–0.54)	
Normal range	42	51.2	40	48.9	1.0	
Excess weight	146	67.9	69	32.1	0.66 (0.49–0.88)	
Waist circumference						0.005
Normal	89	77.4	26	22.6	1.0	
Elevated	143	61.6	89	38.4	1.69 (1.17–2.47)	

95% CI: 95% confidence interval; BMI: body mass index; DM: Diabetes mellitus; PR: prevalence ratio; SAH: systemic arterial hypertension.

**Table 4:** Factors associated with elevated VAT/SAT ratio among outpatients from a university cardiology hospital (*N* = 347).

Variables	Elevated VAT/SAT ratio				PR (95% CI)	*P*
	No		Yes*			
	n	%	n	%		
Sex						0.957
Male	78	66.7	39	33.3	1.01 (0.73–1.38)	
Female	154	67.0	76	33.0	1.0	
Age in years						<0.001
20–59	222	74.5	76	25.5	1.0	
≥60	10	20.4	39	79.6	3.12 (2.45–3.97)	
Race and ethnicity						0.069
White	85	75.2	28	24.8	1.0	
Black	48	64.0	27	36.0	1.45 (0.94–2.26)	
Mixed race	99	62.3	60	37.7	1.52 (1.04–2.22)	
Years of study						<0.001
≤9	27	35.5	49	64.5	2.68 (2.05–3.51)	
>9	204	75.6	66	24.4	1.0	
Income						0.002
Lowest tertile	166	72.2	64	27.8	0.63 (0.47–0.84)	
2nd and 3rd tertile	64	55.7	51	44.3	1.0	
Social status						0.126
A1 e B1 (High)	18	75.0	6	25.0	1.0	
B2 e C1 (Middle)	129	70.5	54	29.5	1.18 (0.57–2.44)	
C2. D e E (Low)	80	60.6	52	39.4	1.58 (0.76–3.25)	
SAH						<0.001
No	148	80.9	35	19.1	1.0	
Yes	84	51.2	80	48.8	2.55 (1.82–3.57)	
DM						<0.001
No	199	76.5	61	23.5	1.0	
Yes	33	37.9	54	62.1	2.64 (2.01–3.48)	
Alcohol intake						<0.001
No	113	58.2	81	41.8	1.0	
Yes	119	77.8	34	22.2	0.53 (0.38–0.75)	
Physical activity status						0.729
Insufficient (<150 min/week)	61	68.5	28	31.5	0.94 (0.66–1.34)	
Sufficient (≥150 min/week)	167	66.5	84	33.5	1.0	
Smoking						0.010
Current	5	45.5	6	54.5	1.87 (1.06–3.31)	
Non-smoker	192	70.8	79	29.2	1.0	
Former	35	53.8	30	46.2	1.58 (1.15–2.18)	
BMI (kg/m^2^)						<0.001
Underweight	40	80.0	10	20.0	1.17 (0.56–2.43)	
Normal range	68	82.9	14	17.1	1.0	
Excess weight	124	57.7	91	42.3	2.48 (1.50–4.10)	
Waist circumference						<0.001
Normal	96	83.5	19	16.5	1.0	
Elevated	133	58.1	96	41.9	2.54 (1.64–3.93)	

^*^Sex-stratified highest tertile (males ≥ 3.60, females ≥ 2.14). 95% CI confidence interval; BMI: body mass index; DM: Diabetes mellitus; PR: prevalence ratio; SAH: systemic arterial hypertension.

After adjusting for confounders, only physical inactivity and elevated WC remained associated with VAT accumulation, with an odds ratio of 2.3 (95% CI 1.1–4.7; *p* = 0.023) for visceral obesity in sedentary individuals and 6.4 (95% CI 2.6–15.8; *p* < 0.001) for individuals with elevated waist circumference. Regarding subcutaneous obesity, older adults, individuals with malnutrition, and those with BMI >25 kg/m^2^ were protected against SAT accumulation, while alcohol consumption increased the odds of SAT accumulation by 2.2 times (95% CI 1.3–3.7; *p* = 0.005), and elevated waist circumference increased this likelihood by 4.5 times (95% CI 2.1–9.8; p < 0.001). An elevated VAT/SAT ratio was more common among older adults (OR 5.5; 95% CI 2.0–14.8; *p* = 0.001), individuals of Mixed Race and Black, those with lower education (OR 2.4; 95% CI 1.1–5.2; *p* = 0.028), and diabetics (OR 2.4; 95% CI 1.2–4.9; *p* = 0.017). Alcohol consumption provided protection against an elevated VAT/SAT ratio (OR 0.5; 95% CI 0.2–0.9; *p* = 0.015) (Table 5).

**Table 5:** Logistic binary regression.

Variables	OR _adjusted_	95% CI	*P* ^*^
Visceral obesity			
Insufficiently active	2.3	1.1–4.7	0.023
WC elevated	6.4	2.6–15.8	<0.001
Subcutaneous obesity			
≥60 years	0.1	0.1–0.5	0.003
Alcohol intake	2.2	1.3–3.7	0.005
Underweight	0.3	0.1–0.8	0.014
Excess weight	0.4	0.2–0.8	0.011
WC elevated	4.5	2.1–9.8	<0.001
VAT/SAT ratio			
≥60 years	5.5	2.0–14.8	0.001
Black	2.7	1.2–6.0	0.017
Mixed race	2.0	1.1–4.1	0.048
Lower educational level (≤9 years of study)	2.4	1.1–5.2	0.028
DM	2.4	1.2–4.9	0.017
Alcohol intake	0.5	0.2–0.9	0.015

^*^Wald Test. 95% CI: 95% confidence interval; BMI: body mass index; DM: diabetes mellitus; OR: odds ratio; SAH: systemic arterial hypertension. Factors associated with visceral, subcutaneous obesity and elevated VAT/SAT ratio.

## 4 Discussion

This study aimed to identify predictive factors for visceral and subcutaneous obesity phenotypes and characteristics associated with an elevated VAT/SAT ratio, demonstrating that distinct factors may influence the accumulation of each abdominal adipose tissue (AAT) component. Our main results demonstrate that physical inactivity was predictor of higher VAT, while older age and alcohol consumption were independently associated with greater SAT accumulation. The predisposition to accumulate VAT, represented by the VAT/SAT ratio, was influenced by sociodemographic factors (older age, non-White race, and lower education) and diabetes. WC was an independent predictor of both VAT and SAT, though not of the ratio between these components.

Studies investigating modulating factors of the abdominal fat distribution across visceral and subcutaneous depots remain underexplored. However, it is known that abdominal fat distribution is influenced by complex interactions among multiple factors, including genetics, sex, race, age, dietary habits, physical activity level, comorbidities, and hormonal factors (23, 26–29). Given the distinct metabolic and functional behaviors of different abdominal fat compartments (30, 31), there is growing global interest in quantifying abdominal adiposity and identifying factors influencing its accumulation (26, 32).

### 4.1 Behavioral factors

In our study, physical inactivity was a predictor of higher VAT accumulation. This result was also found in previous studies (33, 34). Better physical activity status and reduced sedentary behavior favor a negative energy balance by increasing resting energy expenditure. Although the direct causal relationship between physical activity status and preferential VAT reduction remains unclear, evidence suggests that visceral adipocytes are more sensitive to catecholamine stimulation released during exercise than abdominal subcutaneous adipocytes, resulting in greater lipolytic capacity and attenuation of VAT accumulation (27, 33).

The level of physical activity in our study was assessed using the IPAQ; however, only the total time spent on weekly activities was considered in the analysis, without evaluating exercise intensity or sedentary behavior duration. Future research should delve deeper into the role of physical activity in abdominal adipose composition, incorporating data on the frequency and intensity of physical activity. Understanding how different types of daily activities are linked to different adiposity phenotypes could shed light on the mechanisms by which sedentary time and physical inactivity contribute to multiple adverse health outcomes, including changes in body composition. This knowledge could ultimately inform more targeted guidelines on sedentary behavior and physical activity (34).

Lifestyle factors (i.e., behavioral) evaluated in this study differentially influenced AAT components. While physical inactivity influenced VAT accumulation, alcohol consumption modulated SAT accumulation. The role of alcohol consumption in determining AAT components has not been thoroughly explored, and differing results have been reported (35). Some studies identified alcohol as an independent predictor of VAT accumulation (36, 37). These discrepancies may be related to methodological variations in defining “alcohol consumption” or to synergistic characteristics that could amplify or reduce this association’s effect. It’s relevant noting that we did not assess the frequency and intensity of alcohol consumption, which may limit more definitive interpretations and conclusions.

### 4.2 VAT/SAT ratio

Elevated WC was predictive of higher VAT and SAT concentrations but not of an elevated VAT/SAT ratio. This finding underscores WC as a useful screening tool to estimate excessive intra-abdominal fat but highlights its limited ability to discriminate a greater predisposition for VAT over SAT accumulation. Evidence suggests that the VAT/SAT ratio may offer a better metric for assessing cardiometabolic risk than absolute quantification of each depot (12, 38). This is because the ratio provides an estimate of the relative contribution of visceral adipose tissue to total abdominal fat (38).

The ectopic fat model, represented by the VAT/SAT ratio, suggests that excess energy resulting from an imbalance between dietary intake and caloric expenditure is initially stored in subcutaneous compartments. When these subcutaneous stores reach their maximum capacity, the excess energy may be redirected to visceral compartments. This overload of fat in adipocytes can lead to reduced subcutaneous fat storage capacity, resulting in visceral fat accumulation (12). Given the variability in body shape and size across populations, the absolute values of VAT may not adequately reflect the risk differences associated with visceral obesity. In this context, assessing the VAT-related risk becomes challenging in individuals with diverse body types. Taken together, the VAT/SAT ratio theoretically provides a more accurate indicator for evaluating an individual’s body composition and associated health risks.

The VAT/SAT ratio, as measured by US scans, is associated with abnormal glucose metabolism and an increased risk of developing type 2 diabetes mellitus (39, 40). In a cohort of 473 female patients, the VAT/SAT ratio was independently linked to clusters of cardiometabolic risk factors (41). Furthermore, it holds prognostic significance as a unique predictor of cardiometabolic risk, independent of age and BMI (42). However, an elevated SAT may lower the absolute value of the VAT/SAT ratio without mitigating the associated risk. In this context, it is evident that abdominal fat accumulation, irrespective of its specific compartment, poses a health risk and should be addressed as a significant cardiometabolic risk factor.

### 4.3 Biological factors

Notably, aging is associated with changes in body fat distribution, including increases in intra-abdominal fat. Our findings indicated a higher predisposition for VAT accumulation (elevated VAT/SAT ratio) in older individuals (≥60 years). This trend may be explained by age-related body fat redistribution and decreased basal energy expenditure, leading to a greater concentration of visceral abdominal fat, while subcutaneous fat tends to decline (2, 43).

The observation that an elevated VAT/SAT ratio was more common among non-White individuals (Mixed race and Black) may reflect the influence of socioeconomic, biological, environmental, and cultural factors (44), as well as genetic and epigenetic factors (8, 45). However, contrasting with our study, it is generally recognized that African-American men tend to have lower VAT concentrations than White men, whereas VAT levels are more comparable between White and African-American women (46, 47).

The observed differences can be partially attributed to the unique characteristics of our population, including the mixed genetic background of Brazilian individuals, which stems from the integration of Indigenous, European, and African ancestry (48, 49). This high degree of miscegenation presents a challenge when comparing Brazilian individuals with other racial and ethnic groups. Further studies are necessary to investigate the predictive factors of abdominal adipose composition in such a diverse population.

### 4.4 Clinical factors

Demerath et al. (46) showed that SAT concentration may be higher in Black women compared to White women, a difference not observed among men. The independent association of a higher VAT/SAT ratio with lower educational attainment may reflect risk behaviors associated with limited social conditions. Education is a recognized proxy for socioeconomic status, which is a strong determinant of health behaviors across both sexes and all age groups (28). Furthermore, dietary behaviors are influenced by social and educational status, with low socioeconomic and educational levels being associated with obesity and higher consumption of ultra-processed foods and refined carbohydrates (50, 51). Another study highlighted that higher socioeconomic status was linked to healthier dietary choices, particularly more frequent fruit and vegetable consumption (52), a habit that may support a healthier pattern of intra-abdominal fat distribution (2).

### 4.5 Sociodemographic factors

Moreover, the quality of self-care and the ability to interpret information related to preventive health behaviors, including abdominal fat accumulation, can also be influenced by educational attainment and socioeconomic factors (44). However, the relationship between the predisposition for VAT accumulation and socioeconomic status requires further investigation due to the complexity of understanding how social factors may impact biological processes. The association between the VAT/SAT ratio and DM may be bidirectional (53). Excess visceral adiposity can precede the development of DM due to the direct supply of free fatty acids and inflammatory adipokines to the liver, which are secreted by visceral adipocytes. Free fatty acids inhibit insulin secretion from pancreatic cells and limit insulin-induced glucose uptake, likely by impairing signaling and transduction mechanisms (6, 8). Conversely, individuals with DM are at increased risk of developing obesity due to insulin resistance, which raises hepatic glucose production and, consequently, insulin levels, further contributing to fat accumulation (53).

### 4.6 Future perspectives

This study is not without limitations. The sampling method did not allow for a randomized sample, and recruiting individuals from a healthcare setting may limit the generalizability of the findings. The observational design and cross-sectional sample restrict the ability to infer causality from the observed associations. Furthermore, some important variables that influence abdominal adipose tissue composition, such as dietary intake, genetic, and hormonal factors, were not analyzed.

We also acknowledge that the use of more precise instruments could provide more relevant insights into the sociodemographic and behavioral profile of the studied sample; however, this was not feasible in our study. Nonetheless, we emphasize that this limitation reflects the real-world challenges faced in clinical routines within public healthcare settings, where time for detailed investigations is often constrained. Additionally, the absence of analyses using raw/continuous data may limit the modeling effects and the detection of small statistical differences, which we propose as a focus for future investigations.

On a positive note, this study included the use of an imaging technique (i.e., US) for the non-invasive assessment and separate quantification of abdominal fat depots. US has been reported as a useful alternative to reference methods for evaluating different body compartments. Furthermore, we incorporated a wide range of explanatory variables in the conceptual model and assessed the predisposition to accumulate VAT relative to SAT (VAT/SAT ratio). In addition, the intra- and inter-observer calibration evaluation confirmed the adequate reproducibility of the imaging method used, reinforcing the methodological standardization of the study.

It is noteworthy that adipose distribution patterns vary across ethnic groups, highlighting the need for future multicenter, multiethnic studies with large sample sizes. Longitudinal studies are also required to explore the effects of social, biological, and behavioral aspects on adipose characteristics over time. Furthermore, in-depth investigations into the VAT/SAT ratio metric and its metabolic implications should be conducted across different ethnic and social populations.

## 5 Conclusion

Patterns of adipose tissue distribution across different abdominal fat compartments is influenced by complex interactions among multiple factors. Physical inactivity emerged as an independent predictor of the visceral obesity phenotype, while alcohol consumption was associated with a subcutaneous abdominal obesity pattern. DM and sociodemographic factors, such as older age, non-White race, and lower education, were predictive factors for an elevated VAT/SAT ratio. Our study adds to the growing body of evidence, aiding in identifying characteristics that determine different obesity phenotypes, provide relevant data that can guide strategies aimed at groups at potential risk for complications related to the accumulation of TAV, in addition to signaling indicators that can serve as tools for monitoring and evaluating specific provisions for this audience. However, further evidence from diverse populations is still needed to clarify how sociodemographic and behavioral factors influence the accumulation of different abdominal adipose tissue components, considering additional aspects such as dietary intake, genetic, and hormonal factors.

## Data Availability

The original contributions presented in the study are included in the article/supplementary material, further inquiries can be directed to the corresponding author.
